# Raman Mapping as an Investigative Tool for Understanding the Origin of Silica Sphere-like Structures from a Presalt Carbonate Reservoir of the Aptian Barra Velha Formation in the Santos Basin

**DOI:** 10.1021/acsomega.5c06632

**Published:** 2025-10-23

**Authors:** Lenize F. Maia, Rafael de Oliveira, Linus Pauling F. Peixoto, Gabriel A. Barberes, Dalva A. L. Almeida, Flávia C. Marques, Julliana F. Alves, Antonio Carlos Sant’Ana, Celly M. S. Izumi, Gustavo F. S. Andrade, Dorval C. Dias Filho, Delano M. Ibanez, Luiz Fernando C. de Oliveira

**Affiliations:** † 28113 Universidade Federal de Juiz de Fora, Rua José Lourenço Kelmer s/n, Martelos, Juiz de Fora, Minas Gerais 36036-330, Brazil; ‡ Centro de Pesquisas, 42506 Desenvolvimento e Inovação Leopoldo Américo Miguez de Mello (CENPES) PETROBRAS, Av. Horácio Macedo, 950, Ilha do Fundão, Rio de Janeiro, Rio de Janeiro 21941-915, Brazil

## Abstract

Silicification in carbonate deposits refers to a diagenetic process in which silica replaces carbonate minerals, which are typically associated with hydrothermal fluids. During silica deposition under hydrothermal conditions, quartz crystallites often form on the surfaces of microorganisms, acting as nucleation sites. This silica replacement involves the simultaneous chemical transformation of the original skeletal materials, followed by precipitation. Organic matter-filled silica sphere-like structures were identified in the thin sections from the Barra Velha Formation chert (Santos Basin, Brazil), by the use of the Raman spectroscopy mapping technique and Scanning Electron Microscopy (SEM). The remaining organic matter showed two typical bands in the first-order Raman spectrum, i.e., the D-band located at approximately 1350 cm^–1^ and the G-band at about 1600 cm^–1^. The analysis of Raman parameters such as G-FWHM­(Full Width at Half-Maximum of the G-band) and RBS (Raman Band Separation) led to inference of the thermal maturity of samples by calibration against vitrinite standards. Differences in the spectral profile of organic matter obtained with 532 and 632.8 nm excitation radiations revealed the richness of chemical signatures preserved as sphere-like from the pre-salt deep-water Tupi/Lula Field. The SEM analysis revealed silica microsphere morphologies as indicative of the presence of microfossils in this rock. The chemical composition of silica sphere-like structures in BVF may be interpreted as biosignatures preserved in the chert.

## Graphical abstract

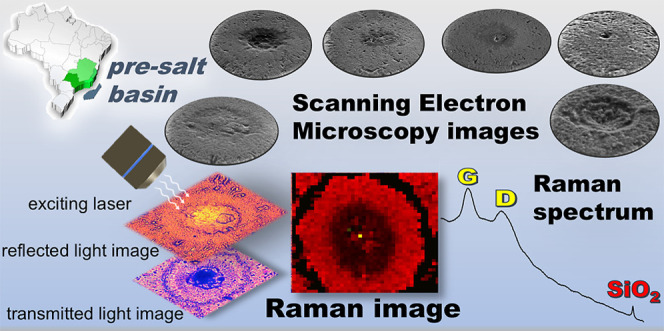

## 1 Introduction

Silicification of carbonate rocks involves the replacement of carbonate by silica and the precipitation of pore-filling silica (SiO_2_) cement.^1^ This diagenetic process is commonly observed in the Barra Velha Formation (BVF), an Aptian presalt unit of the Santos Basin, southeastern Brazil. The authigenic quartz (or silica) formation, particularly in Phanerozoic successions, is often associated with siliceous organisms, whose decay mediates silica nucleation and preservation of biosignatures.^2^ These rocks were interpreted as transitional deposits influenced by microbial activity between continental and marine conditions.^3^ The major facies recognized in the BVF are fascicular calcite crusts (shrubs), Mg-claystones with spherulites, laminites, and intraclastic grainstones.^4,5^ However, these carbonate rocks undergo processes of silicification and dissolution, which can increase or reduce the poro-perm properties of these reservoirs, influencing the quality and fluid flow behavior.^3,6^ Silica in the BVF displays four main diagenetic phases: (1) cryptocrystalline silica; (2) microquartz; (3) fibrous microquartz; (4) megaquartz.^4,6^ The majority of the silica in presalt deposits has been attributed to late diagenetic processes^7–9^ or hydrothermal activities.^5,6^

Microquartz and chalcedony in BVF have always been associated with macroquartz, suggesting a mixed origin of the high silica content by dissolution of Mg-clay and hydrothermal action.^10^ Silica polymorph precipitation involves a series of geochemical changes triggered by the decomposition of organic matter.^11,12^

The mechanism of natural siliceous deposition by hydrothermal events has been proposed experimentally by biotic and abiotic processes,^13–21^ and it has become relevant for identifying their impact on reservoir characterization. Artificial fossilization experiments have revealed the transformations of silica under hydrothermal conditions (elevated temperature and pressure) in which quartz crystallites commonly nucleate on the surface of microorganisms. Literature has demonstrated that spherical morphologies have been interpreted as possible products of microbial silicification, supporting the notion that biosignatures preserved in chert are not unusual phenomena.^22,23^ Authigenic nucleation has been reported in both intact filaments and organic debris derived from the decomposition of cyanobacteria,^14,15,24^ from bacteria,^18,21,25^ microbial biofilms,^26,27^ and archaea.^19,20^ These studies showed that different microbes present species-specific patterns of silicification and that the synthetic quartz crystallizes mainly in the form of radially fibrous spheres. The terminological inconsistency in the nomenclature of silicic spheres accounts for the diverse array of terms used to describe them, such as quartz spherulites,^15^ silica spherulites,^28^ silicate spherulites,^29^ microspheres,^17^ spheroids,^30^ mineral microfossils, spheroid formation,^31^ siliceous ooze,^16^ silicified microfossil,^20^ and organic spheres.^23,32^ In contrast, radial calcite spheres within a micrite matrix are unequivocally termed calcite spherulites. Similar to silica spheres, calcite precipitation in the form of spherulites may occur through organomineralization;^33^ however, the carbonate facies do not typically preserve microbial textures or microfossils as chert-rich facies.^23^

Microfossiliferous chert in the BVF preserved distinct, organic-rich structures and textures according to Moore et al. (2024; 2025).^23,34^ The authors reported that the composition and morphologies of silicified organic structures, namely, organic spheres, are likely primary organic matter and a microfossil-like assemblage. Energy-dispersive X-ray spectroscopy (EDS) maps revealed distinct organic matter forms: (i) clots of organic matter; (ii) diffuse, clotted organic textures; (iii) wispy, branching textures; and (iv) a morphologically diverse group of spherical organic-rich structures that may represent microfossils.^23,34^ Using Ptychographic X-ray-Computed Tomography (PXCT), it was possible to characterize silica–organic relationships, microfossil morphologies, and taphonomic variability.^34^ The preservation of different organic spheres in the chert provided evidence that diverse microbiota thrived in at least some microenvironments in the basin.

In this work, the organic matter content in the silica sphere-like structure from the Tupi Field (Santos Basin) was analyzed by Raman spectroscopy mapping and Scanning Electron Microscopy (SEM). Raman mapping, as an in situ technique, provided information about the chemical composition across the surface of the microsphere, and the morphology revealed by SEM. Raman spectroscopy has been commonly applied to investigate carbonaceous biosignatures in the early rock record^30,32,35–45^ and to characterize the thermal maturity of organic matter (OM) in sedimentary and metamorphic rocks.^46–48^ Raman spectral patterns have been successfully correlated with Vitrinite Reflectance (VR) to determine the thermal maturity of OM based on diverse spectral parameters associated with the first-order G- and D-Raman bands (1800–1100 cm^–1^).^46–50^ The prediction of thermal maturity is based on calibration with Raman spectra of standard samples, such as vitrinite with known % Ro. The estimation of a reflectance equivalent is based on Raman spectral parameters (% *R*_Raman_). It should be noted that Raman spectroscopy and SEM measurements were performed without any additional treatment of the samples, which kept all their characteristics associated with the sampling used in the regular procedures of the industry, indicating that those techniques may be useful to understand the geological samples without any changes to the usual procedures of sample preparation.

The chemical characterization and thermal maturity estimation of diverse silica sphere-like forms shed new light on the origin of organic-filled silica structures in the relevant Santos Basin from the Tupi Field under exploration. The occurrence of organic-rich silica sphere-like structures in BVF chert, characterized by low thermal maturity, provides novel insights into early diagenetic microbial processes in this unique presalt setting.

### 1.1 Geological Framework

The Santos Basin, a passive margin basin on the southeastern Brazilian coast, was formed during the Neocomian rifting of Gondwana.^51,52^ Its tectono-stratigraphic evolution comprises Rift, Post-Rift (Sag), and Drift supersequences deposited over thinned continental crust.^53^ The Aptian stage within this evolution is particularly significant, hosting the prolific Pre-Salt petroleum system, which includes the coquinas of the Itapema Formation (late rift) and, critically, the overlying carbonates of the Barra Velha Formation (early postrift/sag).^53,54^

The Barra Velha Formation, a primary Pre-Salt reservoir, was deposited predominantly during the Aptian (Alagoas Stage) in an extensive, shallow, alkaline, and evaporitic lacustrine system characterized by waters rich in silica and magnesium.^55^ The authors detail its complex facies architecture, categorizing them into in situ (spherulites and shrubs, interpreted as eodiagenetic growths within mud), microbial (stromatolites), reworked (grainstones, packstones indicating higher-energy littoral zones), and altered (weathering profiles/dolocretes formed during subaerial exposure). The distribution of these facies was heavily influenced by paleotopography and lake-level fluctuations, leading to significant heterogeneity within the formation.^55^

Economically, the complex microbialites and associated facies of the Barra Velha Formation, along with the Itapema coquinas, constitute the giant reservoirs of fields like Tupi/Lula, Sapinhoá, and Búzios.^54^ The Pre-Salt petroleum system relies on organic-rich shales within the Itapema, Barra Velha, or lower Ariri formations as source rocks, with the thick evaporites of the Ariri Formation providing an effective regional seal, trapping vast hydrocarbon accumulations in structural and stratigraphic plays within these unique lacustrine carbonates.^53,56^

## 2 Methodology

### 2.1 Sample Preparation

Rock samples from well A at depths of 5111 (1), 5163 (2), and 5188 (3) m drilled in the Tupi/Lula Field (Barra Velha Formation, BVF) were provided by Petróleo Brasileiro S.A. (PETROBRAS). The original rock sample is glued with super glue and dried with a thermal blower. Then, it is taken to the polisher for face preparation, using water or oil, or in a dry form. After cleaning the face, a quick impregnation with epoxy resin and blue ceres dye is performed, heating the sample to ensure rapid resin drying. The sample is then polished on discs of different grits (220, 500, and 1200) until the face is suitable for mounting. The glass slide is cleaned and glued to the sample face with glue, ensuring the elimination of bubbles and proper fixation. After drying, the slide is cut to start the thinning process, which can be done on the Astera or the Discoplan. The glue thickness is monitored to ensure the quality of the final slide. The final thickness of the slides should be approximately 30 μm.

### 2.2 Raman Spectral Acquisition

The Raman spectra were obtained from sphere-like structures dispersed on thin sections in a Bruker dispersive spectrometer, model SENTERRA, with exciting radiation at 532, 632.8, and 785 nm. Raman maps and individual spectra were acquired using a 50× ULWD objective lens (NA = 0.5), with a 3–5 cm^–1^ spectral resolution and a 50 μm confocal aperture. Diameters from collected areas were ca. 2–3 μm with 532 nm, 3–5 μm with 632.8 nm, and 5–10 μm with 785 nm laser lines. Acquisition parameters (time and number of accumulations) varied per sample and are listed in Table S1. The optical power of laser light on the sample was measured with a THORLABS model PM100USB. Data analysis and graphing were performed using Bruker-OPUS 7.2 and OriginLab 8.0 software. No preprocessing was applied to the individual spectra or Raman maps.

#### 2.2.1 General Mapping Conditions

•Thin section from 5111 m: Raman mapping 320 × 280 μm^2^ (625 points) of the semimicrosphere 5111-A (∼290 μm) was performed with the laser line at 785 nm. Random points (p): 5111-A (6p, 9p); 5111-B (10 μm) (1p) with 532 nm.
•Thin section from 5163 m: Raman mappings 540 × 430 μm^2^ of the silica sphere-like 5163-A and 5163-B (∼600 μm each) with 100 points, respectively, were obtained with the laser line at 633 nm. Random points of the silica sphere-like structures with the laser line at 532 nm: 5163-C (∼30 μm), 9p; 5162-D (∼30 μm), 16p. Random points (p) of the silica sphere-like structures with the laser line at 632.8 nm: 5163-C (∼30 μm), 16p.
•Thin section from 5188 m: Raman mapping of the silica sphere-like 5188-A (∼180 μm): 16 points (25 × 25 μm^2^) and 144 points (67 × 67 μm^2^) with the laser line at 532 and 632.8 nm, respectively. Silica sphere-like 5188-B (∼200 μm): mapping of 33 × 33 μm^2^, 16 points and 30 × 35 μm^2^, 36 points performed with 532 and 632.8 nm, respectively. Silica sphere-like 5188-C (∼200 μm): 200 × 160 μm^2^ with 1600 points with the laser at 633 nm.

### 2.3 Raman Spectral Processing

Preprocessing, such as linear baseline correction and spectrum normalization, was performed only when the deconvolution of bands was considered by using Origin 2018 software. Baseline correction was carried out using the second derivative (zeroes) method, with zero-crossings of the second derivative (after adjacent-averaging smoothing) serving as anchor points. For comparison, a manual correction was also applied by using the line interpolation method, defined by two baseline points at the spectrum edges. Spectral normalization in Origin was performed by scaling intensities relative to the maximum band height so that all spectra are adjusted to a common intensity range (*I*/*I*_max_), enabling direct comparison.

Deconvolution of the OM bands in the range of 1750–1100 cm^–1^ was performed by Fityk 1.3.1 software to separate the overlapping signals. Deconvolution was carried out on preprocessed spectra by fitting a sum of Gaussian functions using nonlinear least-squares minimization through the Levenberg–Marquardt algorithm, with the band position, width, and intensity optimized to reproduce the experimental data, i.e., G- and D-bands.

All Raman chemical maps were generated in OPUS software by integrating selected spectral regions after applying a linear baseline correction defined by connecting the edges of each integration range. For silica, the band was integrated between 485 and 445 cm^–1^, while for the G-band, the integration range was 1620 to 1530 cm^–1^.

### 2.4 Thermal Maturity Raman Parameters

During thermal maturation, organic matter undergoes chemical transformations, and the molecular structure is rearranged from low to higher ordering as its geochemical maturation increases. The Raman spectrum of OM consists of a first-order region (1700–1000 cm^–1^) and a second-order region (3300–2300 cm^–1^).^56^ In this work, we used the first-order region ranging from 1750–1100 cm^–1^, comprising two main bands: the graphite or graphite-like band (G-band) around 1580 cm^–1^ and the disordered band (D-band) ranging from 1360 to 1340 cm^–1^. The G-band arises from single-resonance in-plane E_2g_ vibrational modes where the C–C bonds in the lattice are stretched.^57^ The D-band is associated with structural defects and heteroatoms. It originates from a double-resonance (DR) “radial breathing mode” of the hexagonal carbon rings in the lattice.^57^ According to the molecular model described in Lünsdorf (2016)^46^ and references cited therein, the observed intensity in the D-band region is due to the collective ring breathing vibration, and the position of the D-band is then related to the dimensions of the molecule/subunit. The term “D-band” is used for nondeconvoluted spectra since the D-broad band can be decomposed into several bands. The most common bands are as follows: D1-band (1340 cm^–1^), D2-band (1610 cm^–1^), D3-band (1500 cm^–1^), D4-band (1200 cm^–1^), D5-band (1260 cm^–1^), and D6-band (1440 cm^–1^). Raman parameters such as fwhm—Full Width at Half-Maximum, RBS—Raman Band Separation, and R1—Ratio of Raman band height (*I*_D_/*I*_G_) or area (*A*_D_/*A*_G_) can be applied according to the sample type.^47^ As maturity increases, G-FWHM and R1 decrease, and RBS increases. The prediction of thermal maturity was based on calibration with Raman spectra of vitrinite with a known percentage of reflectance (% Ro) obtained in 532 and 632.8 nm exciting radiations. The correlation between % Ro and G-FWHM can be represented by an exponential function (eq 1 below) previously described by Barberes et al. (2025).^58^ The exponential relationship for 532 nm is expressed in eq 1.

1 $$\%\;Ro=0.53901+98.46014e^{\left(-G\;FWHM/10.04782\right)}$$

## 3 Results and Discussion

The organic matter content in the silica sphere-like structures (Figure 1) from ultradeep waters of Tupi Field (Santos Basin) was analyzed by Raman spectroscopy mapping and Scanning Electron Microscopy (SEM). The characterization of samples from diverse depths follows.

**Figure 1:**
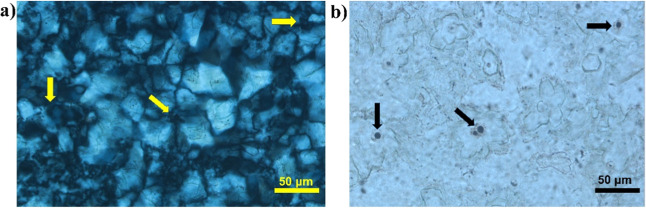
(a) Cross-polarized and (b) plane-polarized thin-section photomicrographs from Tupi Field in Santos Basin (5188 m deep) showing silica sphere-like structures in an interelement pore (intensely recrystallized to microcrystalline quartz) with organic matter in its center (arrows).

### 3.1 Raman Spectral Analysis

Raman spectra were obtained on silica sphere-like structures ranging from 300 to 10 μm in diameter, observed in thin sections at depths of 5111, 5163, and 5188 m. Figure 2 shows a semicircle-shape silica sphere from 5111 m with a size of approximately 300 μm (5111-A), identified by Scanning Electron Microscopy (SEM) (Figure 2a,b). Raman mapping (320 × 280 μm^2^) obtained with laser excitation at 785 nm showed bands at 464 ν­(Si–O), 206 (A_1_), and 128 cm^–1^ *E*(LO + TO) assigned to silicates^59,60^ and two other bands around 1580 (G-band) and 1330 cm^–1^ (D-band) attributed to OM (Figures 2c and S1).^46–50^ Figure 2 shows a SEM (Figure 2a) and Raman optical micrograph (Figure 2b) of the mapped area in the thin section from the sample at 5111 m. The representative Raman spectra attributed to OM and silicate are shown in Figures 2c,d and S1. The chemical map represented by the area of marker bands at 1580 and 465 cm^–1^ can be observed in Figure 2e,f in the heat map, where pink and red colors are associated with the highest signal intensity and dark blue with the lowest. Integration of the band at 1580 cm^–1^ suggests that the OM is present at the semisphere edges, while the band at 465 cm^–1^ is detected predominantly in the area external to the semisphere and to a lesser extent in the internal area. The low intensity of Raman bands in the spectra may be due to the semisphere’s irregular texture (Figure 2a), showing cavities that hinder sample focusing, consequently leading to a loss of signal intensity. Nevertheless, the Raman intensities were also affected by the fluorescence background, which is significantly more intense in the presence of OM, as depicted in Figure 2c. The fluorescence background correction achieved by fitting the baseline to each of the selected spectra (Figure 2d) confirmed the identification of OM and SiO_2_.

**Figure 2:**
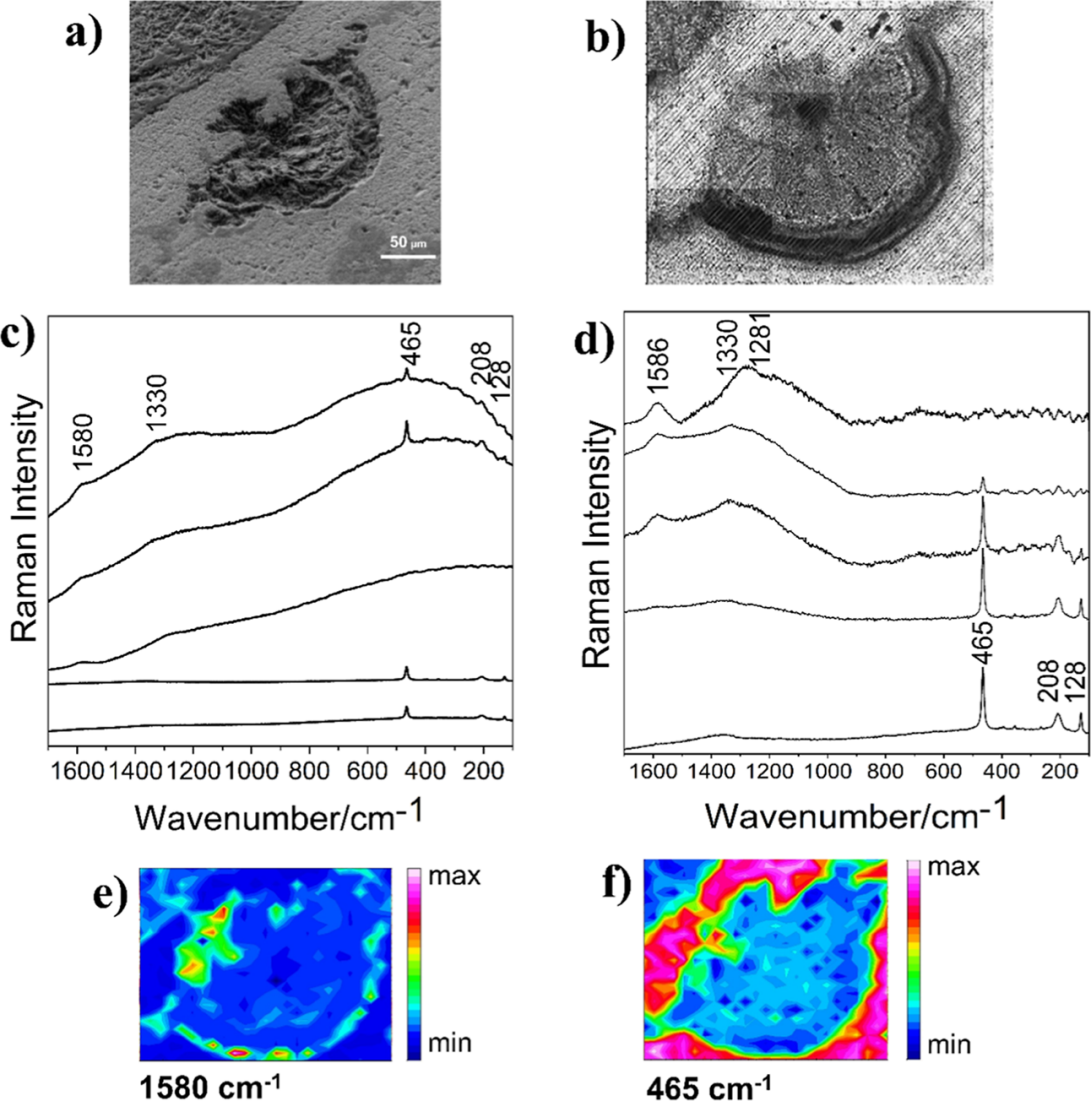
SEM and Raman analyses of the petrograph thin section from a 5111 m depth. (a) SEM at 45° (800×), (b) reflected light imaging of the silica semisphere 5111-A (∼300 μm), 4× magnification, (c) raw spectra, and (d) baseline-corrected spectra obtained from mapping with 625 points (250 × 300 μm^2^) with the excitation laser line at 785 nm, 50 mW, 2 accumulations, 10 s; (e,f) Chemical maps obtained by the integration of the Raman band at 1580 cm^–1^ (G-band) and 465 cm^–1^ (SiO_2_), respectively. The color scale indicates the magnitude of the integrated band area.

The literature has suggested that excitation lines at 532 or 514 nm are more suitable for spectral characterization of OM due to the lower influence of the fluorescence effect.^49^ A detailed examination of the edges and protruding regions performed in the 532 nm excitation line confirmed the presence of OM in the semisphere, as can be seen in Figure 3b,c, with bands around 1600 and 1350 cm^–1^. Raman bands with similar wavenumbers (1600 and 1347 cm^–1^) were also observed in the 10 μm sphere-like shape 5111-B identified in the vicinity (Figure 3d). Greater heterogeneity in spectral patterns was observed, which is compatible with the hypothesis of the microbial origin of these structures.

**Figure 3:**
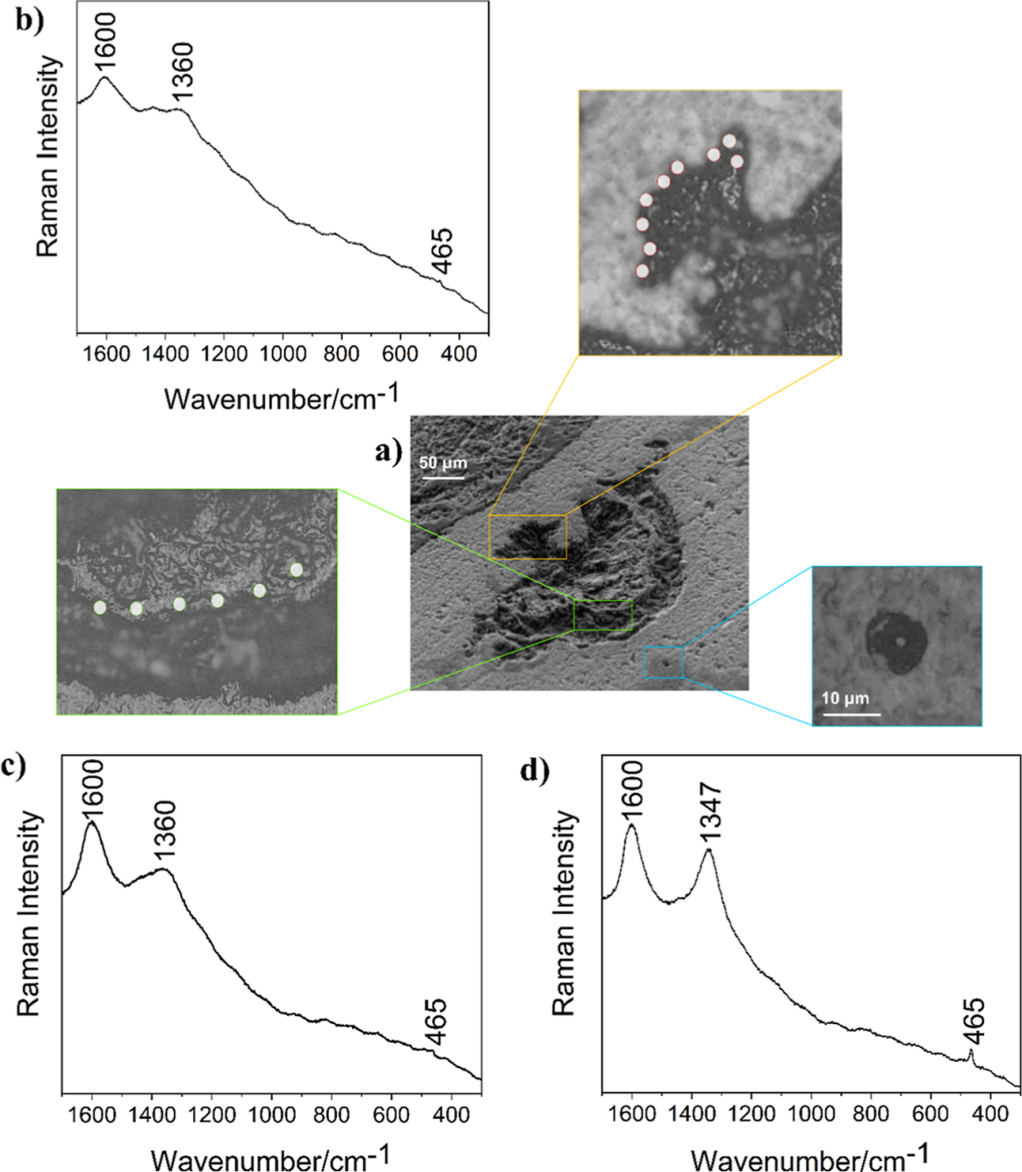
SEM and Raman analyses of the petrograph thin section from 5111 m depth. (a) SEM at 45° (800×), (b,c) average Raman spectra from mapping registered in protruding regions of the silica semisphere 5111-A with the excitation laser line at 532 nm, 1.3 mW, 3 co-additions, 15 s, 10s performed in 9 and 6 points, respectively, and (d) Raman spectrum of 10 μm silica sphere-like 5111-B located next to the larger one with the excitation laser line at 532 nm, 1.3 mW, 3 co-additions, 15 s, 10 s.

At a depth of 5163 m, the silica sphere-like structures with approximately 30 μm are dispersed around the carbonate spherulites (Figure 4), which were identified by the calcite bands at 1086 ν_s_(C–O) and 713 cm^–1^ δ­(O–C–O)^61,62^ registered in Raman maps available in the Supporting Information (Figure S2). Selected sphere-like structures were analyzed at different focal distances with laser radiation at 532 and 632.8 nm. Representative spectra obtained from Raman maps of two silica sphere-like structures are shown in Figure 4. Raman maps obtained from different silica sphere-like showed a similar spectral pattern (Figure S3). The heat maps in Figure 4 show bands at 1590 and 1350 cm^–1^ assigned to OM structures, and at 465 cm^–1^ assigned to silica mode, indicating an even dispersion of the OM in the cavities, as observed in the previous sample. Analysis performed with the same sample at a 632.8 nm excitation (Table S2) showed a slight variation in the Raman wavenumbers, as expected due to different influences of RR effects on OM materials when using distinct exciting radiations.^58^

**Figure 4:**
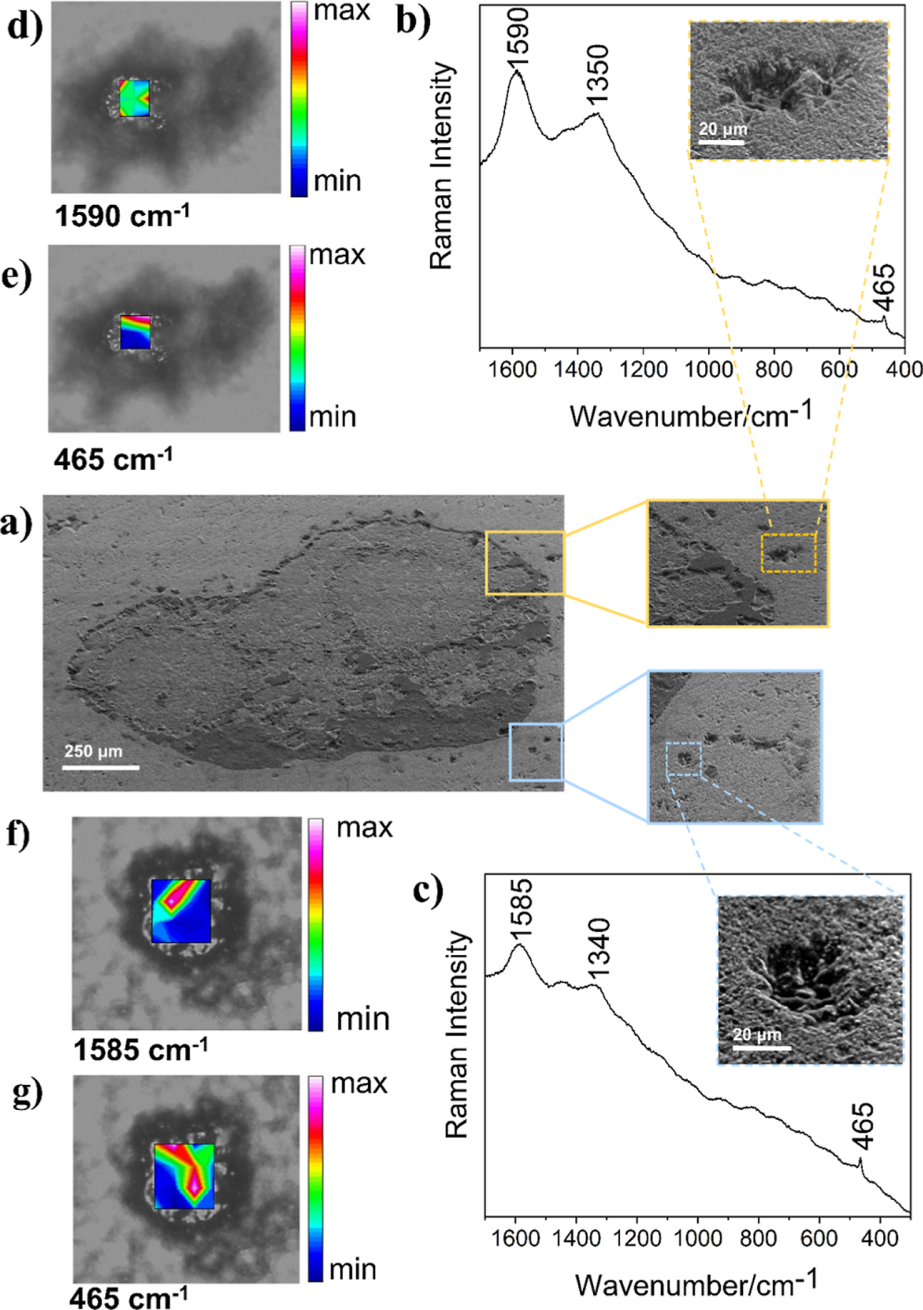
SEM and Raman analyses of the petrograph thin section from a 5163 m depth. (a) SEM at 45° (180×) of carbonate spherulite showing the surrounding silica sphere-like structures; (b,c) selected Raman spectra obtained from mapping performed in 9 and 16 points, respectively, with the excitation laser line at 532 nm, 6.3 mW, 3 accumulations, 10 s. Chemical maps obtained by the integration of the Raman band: (d,f) 1600 cm^–1^ (G-band) and (e,g) 464 cm^–1^ (SiO_2_). The color scale indicates the magnitude of the integrated band area.

The thin section from a depth of 5188 m presented silica spherelike structures with core sizes ranging from 30 to 90 μm. Raman maps obtained with 532 and 632.8 nm laser lines of the sample showed similar spectral patterns with G- and D-bands around 1575 and 1330 cm^–1^ for the 532 nm laser and 1600 and 1325 cm^–1^ for the 632.8 nm laser, respectively (Figure 5). The band assigned to silica mode can be seen at 465 cm^–1^; however, the band at 785 cm^–1^ was attributed to the transverse optical (TO) mode of silicon carbide (SiC),^63^ which may be a contaminant due to sample preparation. Selected spectra are shown in Figure 5c,d. The Raman mapping with 532 and 632.8 nm lasers indicated the occurrence of OM in the central portion of the sphere-like structure.

**Figure 5:**
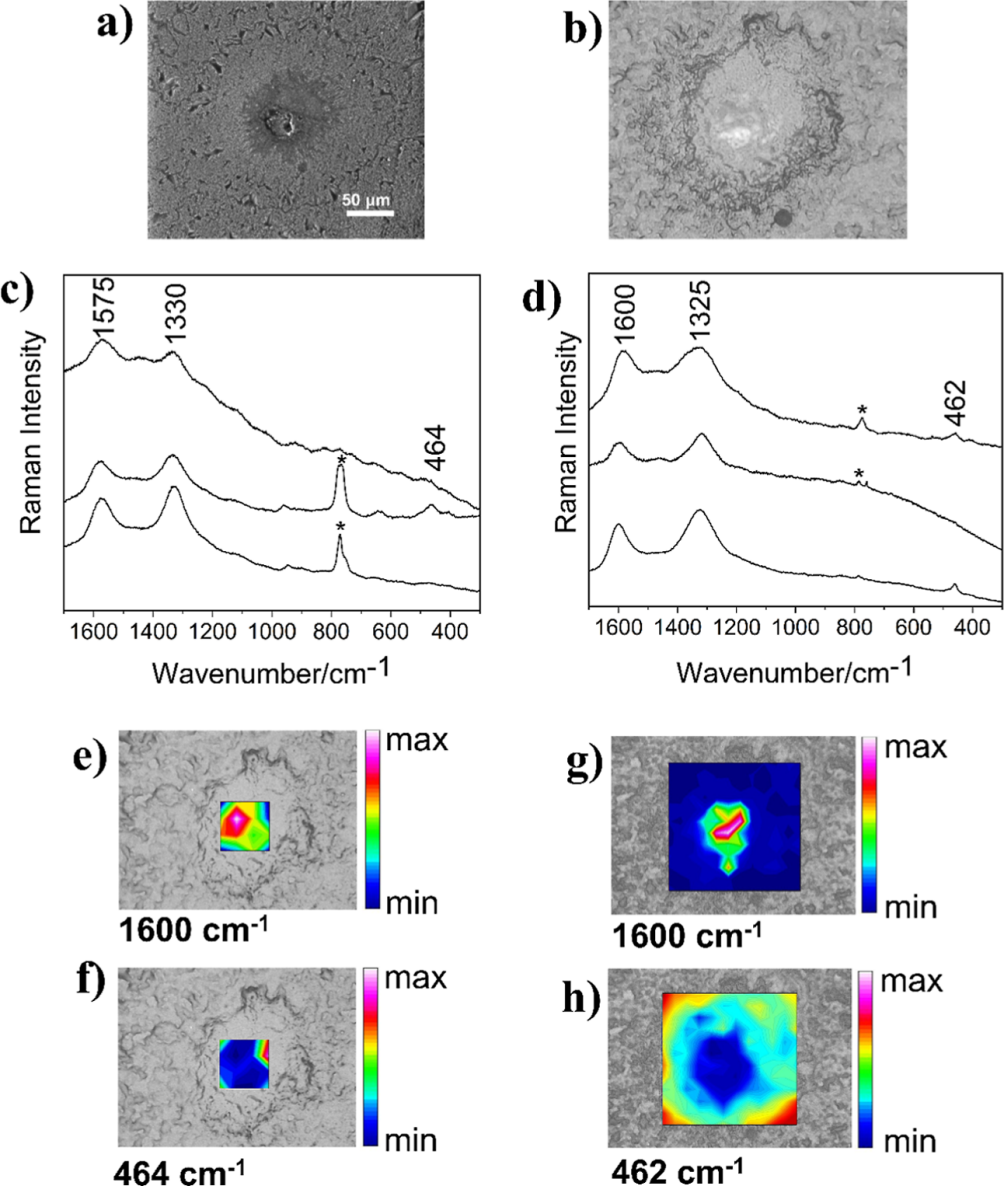
SEM and Raman analyses of the petrograph thin section from a 5188 m depth. (a) SEM with 1200× magnification of the sphere-like 5188-A. (b) Reflected light imaging of the silica microsphere (70 μm), 50×, (c,d) selected Raman spectra obtained from mapping performed with 532 nm (16 points, 6.3 mW, 2 accumulations, 15 s, and 632.8 nm (144 points, 9 mW, 2 accumulations, 15 s), (e,g) chemical maps obtained by the integration of the Raman band at 1580 cm^–1^ (G-band) at 532 and 632.8 nm, respectively, and (f,h) chemical maps obtained by the integration of the Raman band at 465 cm^–1^ (SiO_2_) at 532 and 632.8 nm, respectively. The color scale indicates the magnitude of the integrated band area. Bands marked with an asterisk are attributed to silicon carbide.

OM was also identified in the silica sphere-like 5188-B by the Raman mapping obtained with 532 and 632.8 nm excitation. The analysis showed the G-band in the range of 1580–1570 cm^–1^ and the D-band in the range of 1370–1302 cm^–1^ (Figure 6). The integration of the G-band from spectra obtained with the 532 nm excitation showed a wider dispersion than the integration from spectra with 632.8 nm (Figure 6d,e). These results again indicate significant heterogeneity in the OM spectral patterns, which allows the association of the origin of these sphere-like structures with microorganisms.

**Figure 6:**
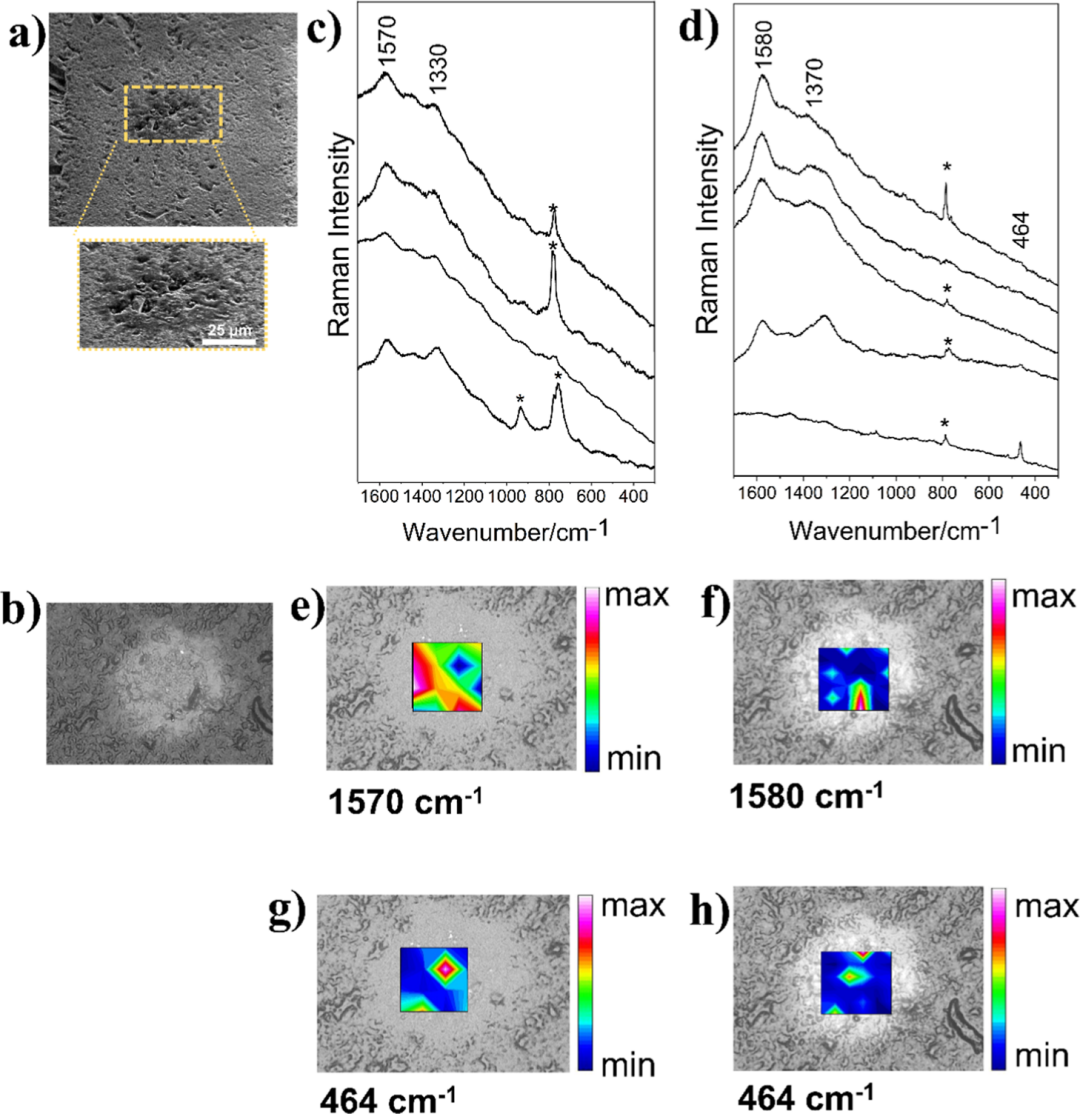
SEM and Raman analyses of the petrograph thin section from a 5188m depth. (a) SEM at 45° (1500×) of the sphere-like 5188-B. (b) Reflected light imaging of the silica sphere-like structure (60 μm), 50×. (c) Selected Raman spectra obtained from mapping (33 × 33 μm) performed with a 532 nm excitation (16 points, 6.3 mW, 2 accumulations, 10 s) and (d) a 632.8 nm excitation (30 × 35 μm, 36 points, 9 mW, 2 accumulations, 15 s). (e,f), Chemical maps obtained by the integration of the Raman band at 1580 cm^–1^ (G-band) at 532 and 632.8 nm, respectively. (g,h) Chemical maps obtained by the integration of the Raman band at 465 cm^–1^ (SiO_2_) at 532 and 632.8 nm, respectively. The color scale indicates the magnitude of integrated Raman bands. Bands marked with an asterisk are attributed to silicon carbide.

The radial area of the sphere-like 5188-C was investigated to evaluate the distinct layers shown in the micrographs (Figure 7a,b). Raman mapping was performed in the area of 270 × 370 μm (1600 points) encompassing a “core” and a “halo” in analogy to the hydrothermal crystallization of silica gel described by Oehler (1976).^15^ Integration of the Raman band at 1598 cm^–1^ revealed the presence of OM in the middle of the sphere-like structure (Figure 6e). The integration of the characteristic band of silicates at 464 cm^–1^ can be seen throughout the sphere-like structure, with different intensities demonstrated in the heat map (Figure 7f). An in-line Raman mapping of the 5188-C sample confirmed the co-occurrence of SiO_2_ and OM within the low-frequency spectral range (Figure S4).

**Figure 7:**
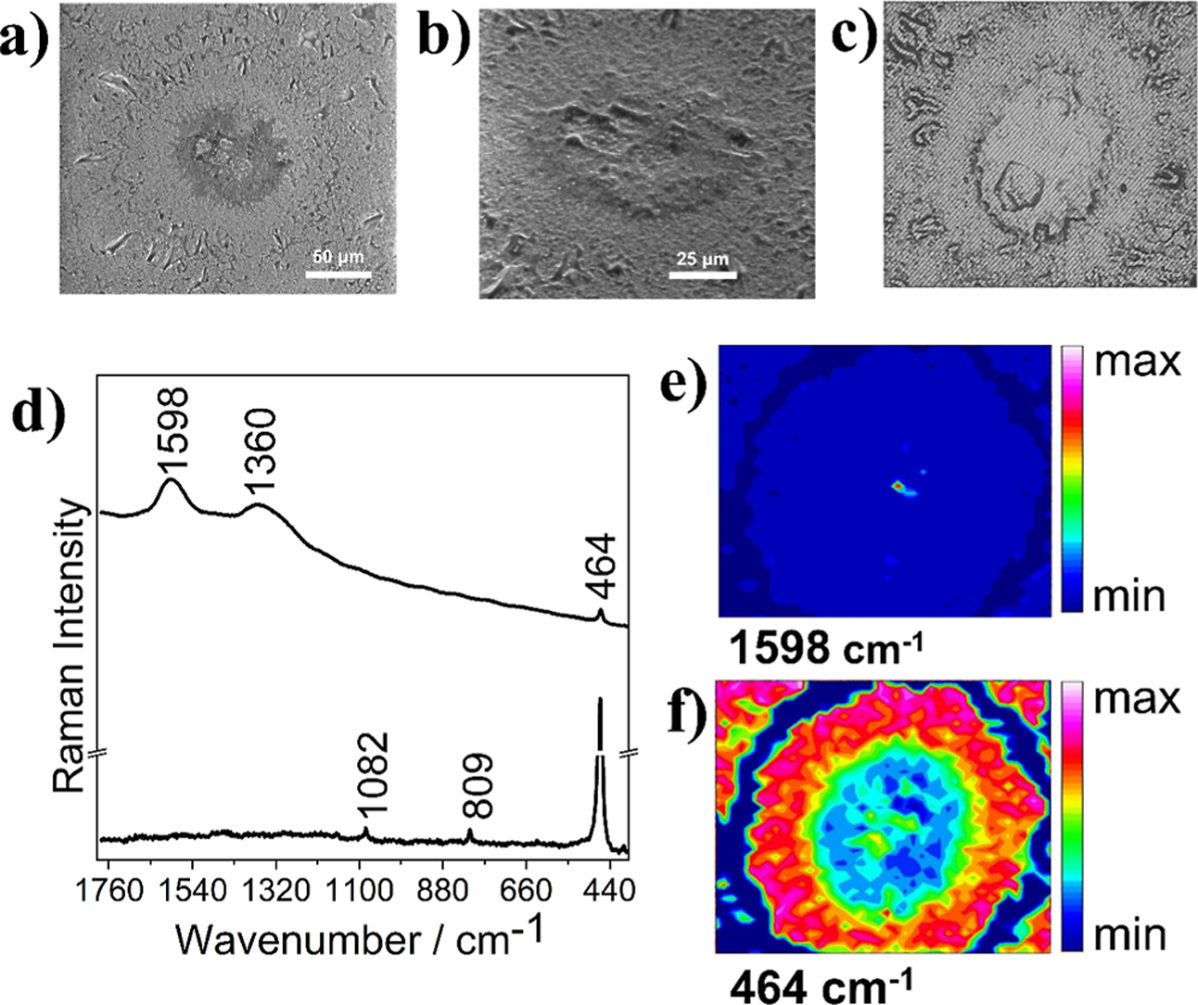
SEM and Raman analyses of the petrograph thin section from a 5188m depth. (a) SEM with 1200× magnification of the sphere-like 5188-C. (b) SEM at 45° (2000×). (c) Reflected light imaging of the silica microsphere (70 μm), 50×, showing the mapped area. (d) Representative Raman spectra obtained from mapping performed with 632.8 nm (1600 points, 12.7 mW, 2 accumulations, 15 s). (e,f) Chemical maps obtained by the integration of the Raman band at 1598 cm^–1^ (G-band) and 464 cm^–1^ (SiO_2_), respectively. The color scale indicates the magnitude of integrated Raman bands.

It is known that silicification can occur as spherical deposits of silica in the groundmass (extracellular polymeric substances) from preserved microorganisms.^21^ The resulting morphology depends on the fossilization time and the amount of silica available.^21,23^ The Raman mappings obtained in the sphere-like structures ranging from 30 to 90 μm showed that OM occurs in the “core” as previously reported in the artificial experiments with several microorganisms.^15,21^ On the other hand, the mapping performed in the semicircle silica sphere 5111-A revealed a wider range of OM dispersion. Differences in the morphology and chemical composition may be attributed to different substrates/organisms. However, the spectral pattern does not differ much from the smaller sphere-like structure (Figure 8).

**Figure 8:**
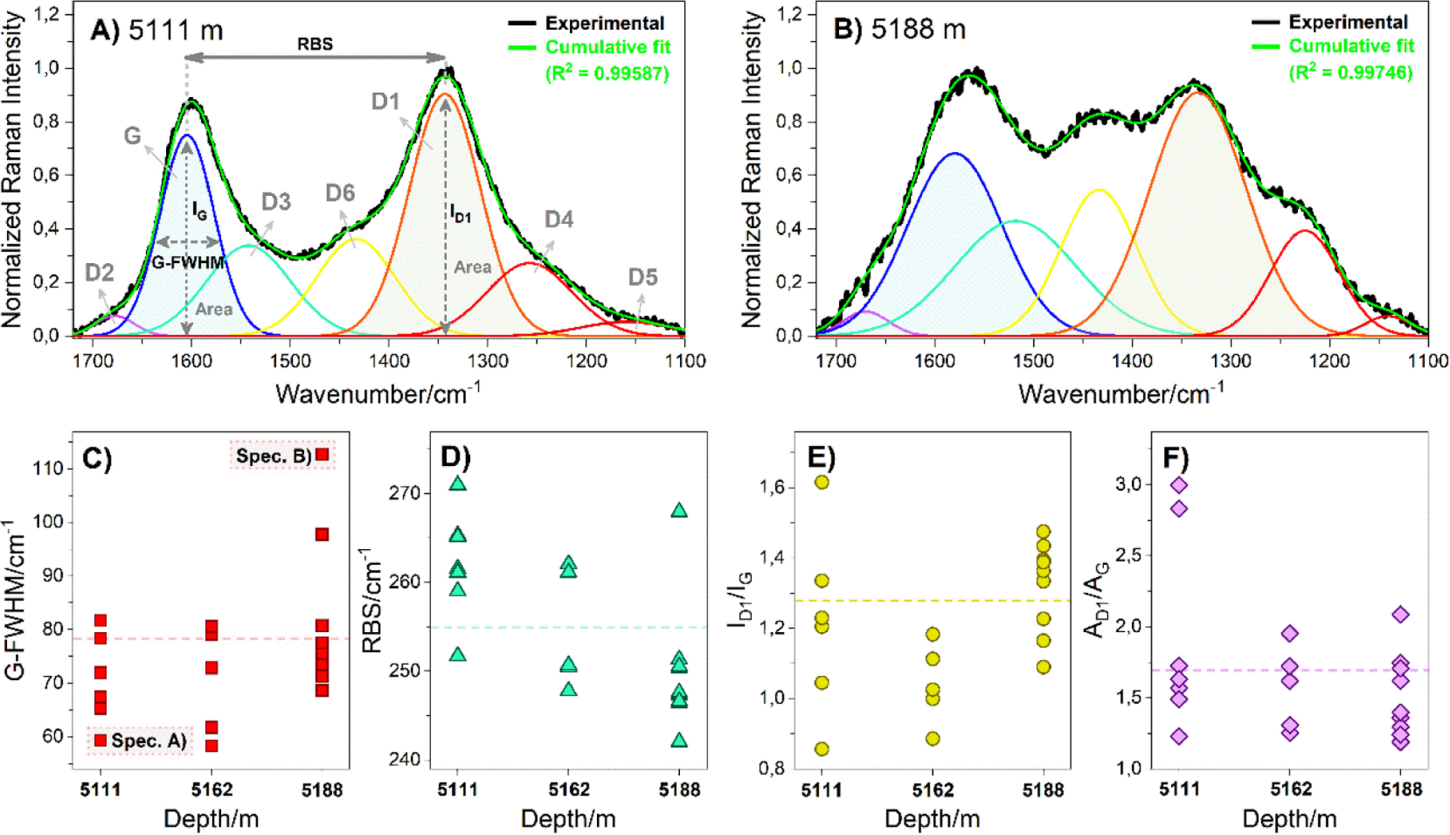
Average Raman spectra and Raman parameters calculated from organic matter identified in silica sphere-like structures. (A) Illustrative deconvoluted spectrum showing the lowest G-FWHM values and (B) the highest G-FWHM values. Correlation of several samples in their respective depths with thermal maturity parameters: (C) G-FWHM, (D) RBS, (E) *I*_D1_/*I*_G_, and (F) *A*_D1_/*A*_G_. The *R* ^2^ values for all spectra were >0.99, and they are listed in Table S2.

The use of several excitation wavelengths (532, 632.8, and 785 nm) enabled the observation of differences in the spectral profile of organic matter related to RR effects. Short linear aromatic systems are in resonance with 532 and 632.8 nm radiation, whereas larger and more compact polycyclic aromatic hydrocarbons are in resonance with the 785 nm line.^64^ Analyses using 532 and 632.8 nm excitation lines showed the OM matrix complexity based on the chemical diversity of each silica sphere-like structure. The presence of distinct Raman spectral patterns from different but nearby silica sphere-like structures at the same depth supports a biotic origin.

### 3.2 Assessment of Thermal Maturity

The Raman line shape of the G-band around 1600 cm^–1^ and the D-band around 1350 cm^–1^ (Figures 2–7) from samples collected at different depths indicated variations in chemical composition among the silica sphere-like structures. This spectral heterogeneity is likely influenced by the origin and preservation state of the OM. During thermal maturation, the OM undergoes significant changes in its structure and composition, leading to observable key features such as the position of the Raman bands (wavenumber or Raman shift), the relative shifting of individual bands, their relative intensities, and the narrowing or broadening of particular bands. Broad bands can be decomposed into several bands, which have been used to evaluate the spectral pattern of OM (Table S2). The method enables decomposing overlapping disordered bands (e.g., D1, D2, D3, D4, D5, and D6) and subsequent calculation of Raman parameters, in comparison with Raman results from calibration samples of vitrinite.^47,49,50^ According to Henry et al. (2019), the G-FWHM and RBS are the parameters most used to estimate the maturity of source rocks in the oil, wet gas, and dry gas generation stages.^47^ Nevertheless, they show an inverse correlation: G-FWHM decreases, while RBS increases with increasing maturity. The G-FWHM is the main parameter used in this work since it has been reported to be unaffected by polishing procedures,^46,65–67^ and presents a good correlation to vitrinite reflectance and its Raman pattern, in sedimentary contexts.^47,68^ The Raman spectra of the OM registered in the spheres-like structures (Figure 8 and Table 1) are characteristic of OM in the coalification process (0.40 to 1.10 Ro %) as described by Schito et al.^50,69^

**Table 1:** Raman Spectral Parameters (532 nm) Calculated from Organic Matter Identified in the Silica Sphere-like Structures

depth/m	spectral code	D1-wavenumber/cm^–1^	G-wavenumber/cm^–1^	G-FWHM/cm^–1^	RBS/cm^–^^1^	*I*_D1_/*I*_G_	*A*_D1_/*A*_G_	estimated % Ro^58^
5111	5111a2	1340.39	1605.53	71.94	265.13	2.07	3.00	0.62
	5111a2	1336.64	1607.60	67.42	270.96	1.23	1.63	0.66
	5111a4	1344.70	1603.74	81.70	259.05	1.04	1.49	0.57
	5111a2	1342.31	1607.66	66.45	265.35	1.62	2.83	0.67
	5111b	1343.06	1604.62	59.38	261.56	1.34	1.73	0.81
	5111a1	1352.17	1603.91	78.44	251.73	0.86	1.23	0.58
	5111b	1343.29	1604.41	65.28	261.13	1.20	1.57	0.69
5163	5163e	1342.31	1604.40	58.32	262.09	1.18	1.95	0.84
	5163f	1338.49	1588.92	72.90	250.43	0.89	1.25	0.61
	5163f1	1341.43	1602.56	61.78	261.13	1.00	1.62	0.75
	5163c	1343.49	1594.18	79.19	250.69	1.11	1.72	0.58
	5163d	1342.99	1590.79	80.56	247.80	1.03	1.31	0.57
5188	5188a	1333.02	1579.48	112.67	246.46	1.33	1.36	0.54
	5188a	1327.77	1578.17	72.54	250.40	1.16	1.19	0.61
	5188a	1329.71	1577.10	80.72	247.38	1.23	1.29	0.57
	5188b	1330.36	1572.40	75.64	242.05	1.36	1.75	0.59
	5188a	1328.22	1579.56	68.64	251.34	1.43	1.62	0.65
	5188a	1332.62	1580.23	77.53	247.60	1.40	1.71	0.58
	5188a	1336.15	1586.69	73.49	250.54	1.09	1.24	0.60
	5188b	1331.01	1577.64	97.79	246.63	1.39	1.40	0.54
	5188c	1339.13	1607.07	71.33	267.94	1.47	2.09	0.62
max		1352.17	1607.66	112.67	270.96	2.07	3.00	0.84
min		1327.77	1572.40	58.32	242.05	0.86	1.19	0.54
average		1338.06	1593.17	74.94	255.11	1.26	1.67	0.63

The heterogeneity of the OM along the depths is highlighted in Figure 8A,B, illustrating the Raman parameters, which display deconvoluted spectra with one of the lowest and highest G-FWHM values, respectively. The spectral pattern differences evaluated by the G-FWHM parameter (Figure 8C and Table 1) suggested the immature-to-mature stage of the OM. Thermal maturity was proposed by the estimated % Ro in the range of 0.54 and 0.84 using the equation described in Section 2.3 and comparison with literature data.^49,50,70–72^ The results obtained from G-FWHM analysis were corroborated by the RBS (Table 1 and Figure 8D), *I*_D_/*I*_G_, and *A*_D_/*A*_G_ parameters (Table 1 and Figure 8E,F).

The molecular dissimilarities shown in Figure 8 are likely a consequence of carbon precursor heterogeneity and subsequent geochemical alterations during postdepositional processes. The structural organization/ordering of OM is related to its thermal maturation, correlated with the biological origin preserved in microfossils.^73^ Silicification preserved many fossils and microbial mats, and the silica replacement is concurrent with the degradation of organic matter.^74^ Moore et al. (2024) demonstrated that the chert in the BVF preserves distinct, organic-rich structures and textures, indicating that this environment was colonized by microbial communities and experienced rapid silica precipitation.^23^ The organic matter preserved in BVF is consistent with diverse morphologies represented by a range of microfossils of bacteria, simple eukaryotes, or possibly pollen.^23^ The characterization of the primary OM using EDS spot analyses revealed a higher oxygen-to-carbon (O/C) ratio than the void-filling thermally mature bitumen found within carbonate facies in the presalt deposits.^23^ The oxygen and silicon isotope analyses measured by secondary-ion mass spectrometry demonstrated that δ^18^O values between 29.1‰ and 41.0‰ and δ^30^Si values between 0.5‰ and 4.5‰ were more consistent with silica precipitation from a low-temperature groundwater or river water source. The authors proposed that microfossiliferous chert in BVF contains one of the earliest generations of silica in the presalt deposits, which preserves kerogen, delicate microbial structures, and microfossils.^23^

The data from the literature were corroborated by the restricted thermal maturity interval (0.54–0.84% Ro), estimated by % Ro equivalent values from Raman parameters (% *R*_Raman_), encompassing the immature to midmature stages of hydrocarbon generation (0.3 < % Ro eq < 1.5).^48,58,69,75^ The low temperature favored the preservation of low-grade thermal degradation of OM in the Tupi Field (Table 1). Our results obtained across well A in BVF showed that the chemical variability of OM in the cavities of the silica sphere-like structures is consistent with higher heterogeneity of the molecular structure in a low-grade thermally mature environment.^76^ This may explain the maturity degree predicted for the silica sphere-like structures along the investigated depths. As previously described for the BVF chert, the confinement of OM in small cavities, the presence of primary organic matter, and diverse microfossil assemblages^23^ support the microorganism origin of the sphere-like structures.

## 4 Conclusion

This work extends the understanding of organic and inorganic chemical species distribution within sphere-like silica structures, with micrometer spatial resolution, preserved in the BVF, using Raman mapping and SEM micrographics. Raman mapping offered a more accurate statistical estimation of the heterogeneity of OM present in these samples at the microscale level compared to single-spectrum acquisition. The use of 532, 632.8, and 785 nm excitation lines showed differences in the spectral profile of the OM related to resonance Raman effects intrinsic to active vibrations. The analyses based mainly on G-FWHM and RBS parameters explored by exciting radiation revealed the richness of OM chemical signatures preserved in sphere-like structures from Pre-Salt deep water around 5000 m. Although Raman spectroscopy did not provide definitive information about the complex OM composition, it enabled the identification of diverse chemical structures correlated with the thermal evolution of OM as a response to increasing burial and time. The silicification as sphere-like structures, concomitant with the occurrence of OM in a nonporous silica structure, could suggest a biotic origin. Therefore, the silica sphere-like morphology could be interpreted as a product of microbial silicification compatible with silica precipitation processes in contact with their structures. Besides, the geometry, size, and Raman spectral heterogeneity may corroborate their genesis. Likewise, the presence of nonporous silica structures around these cavities may suggest that the induction of silica solidification took place around the microorganism, which acted as nucleation sites. In this sense, silica sphere-like in BVF may be a biosignature revealed by the Raman spectral analysis of OM. The thermal maturity interval encompassing the immature to midmature stages was estimated by % Ro equivalent values from Raman parameters. In this way, Raman spectroscopy as an OM thermal maturity indicator can be a predictive probe for oil, wet gas, and dry gas potential in petroleum source rocks and reservoirs.

## Supplementary Material



## Notes

Detailed experimental parameters for the Raman spectral acquisition, data from deconvoluted Raman band assignments and spectral parameters (532 and 632.8 nm) calculated from organic matter identified in the silica sphere-like structures, location where selected Raman spectra were taken in the sample 5111-A, Raman chemical maps of carbonate spherulites, mapped area of the sphere-like structures surrounding the carbonate spherulites (532 nm), and in-line Raman mapping of sample 5188-C, highlighting the low-frequency Raman bands (PDF)
