# Supplementary material for: Raman Mapping as an Investigative Tool for Understanding the Origin of Silica Sphere-like Structures from a Presalt Carbonate Reservoir of the Aptian Barra Velha Formation in the Santos Basin
Source: ACS Omega. 2025 Oct 23;10(43):51309–21. doi: 10.1021/acsomega.5c06632 (PMC12593057; doi:10.1021/acsomega.5c06632)
Supplement: Supplementary file 1 [file ao5c06632_si_001.pdf]

Supporting Information

Raman mapping as an investigative tool for understanding the origin of silica sphere-like structures from a pre-salt carbonate reservoir of the Aptian Barra Velha Formation in the Santos Basin.

Lenize F. Maia[1], Rafael de Oliveira[1], Linus Pauling F. Peixoto[1], Gabriel A. Barberes[1], Dalva A. L. Almeida[1], Flávia C. Marques[1], Julliana F. Alves[1], Antonio Carlos Sant'Ana[1], Celly M. S. Izumi[1], Gustavo F. S. Andrade[1], Dorval C. Dias Filho[2], Delano M. Ibanez[2], Luiz Fernando C. de Oliveira*[1]

(1) Universidade Federal de Juiz de Fora, Rua José Lourenço Kelmer s/n, Martelos, Juiz de Fora, MG, Brazil, 36036-330.

(2) Centro de Pesquisas, Desenvolvimento e Inovação Leopoldo Américo Miguez de Mello (CENPES) PETROBRAS, Av. Horácio Macedo, 950, Ilha do Fundão, Rio de Janeiro, RJ, Brazil, 21941-915.

*Corresponding author
e-mail:luiz.oliveira@ufjf.br

Figures

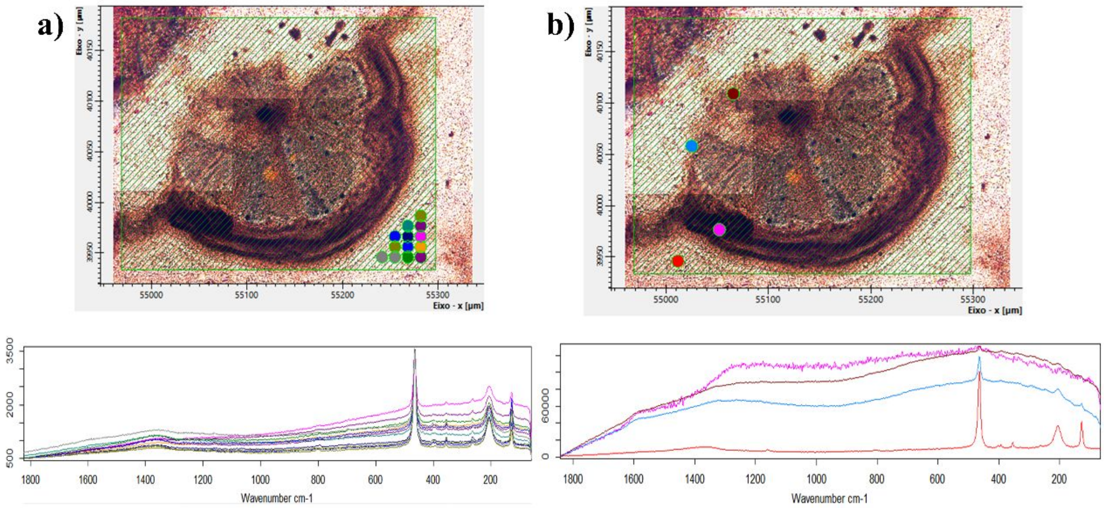

Figure S1. Selected Raman spectra of sample 5111-A obtained from the 625-point (250 × 300 µm) mapping with excitation laser in 785 nm, 50 mW, 2 accumulations, 10 s, with spectral range of 1800-43 cm^{-1}, 3-5 cm^{-1} spectral resolution. Raman spectra corresponding to the colored circle in the reflected light image showing bands assigned to a) Silicon dioxide (SiO_2) and b) organic matter and silicon dioxide (SiO_2).

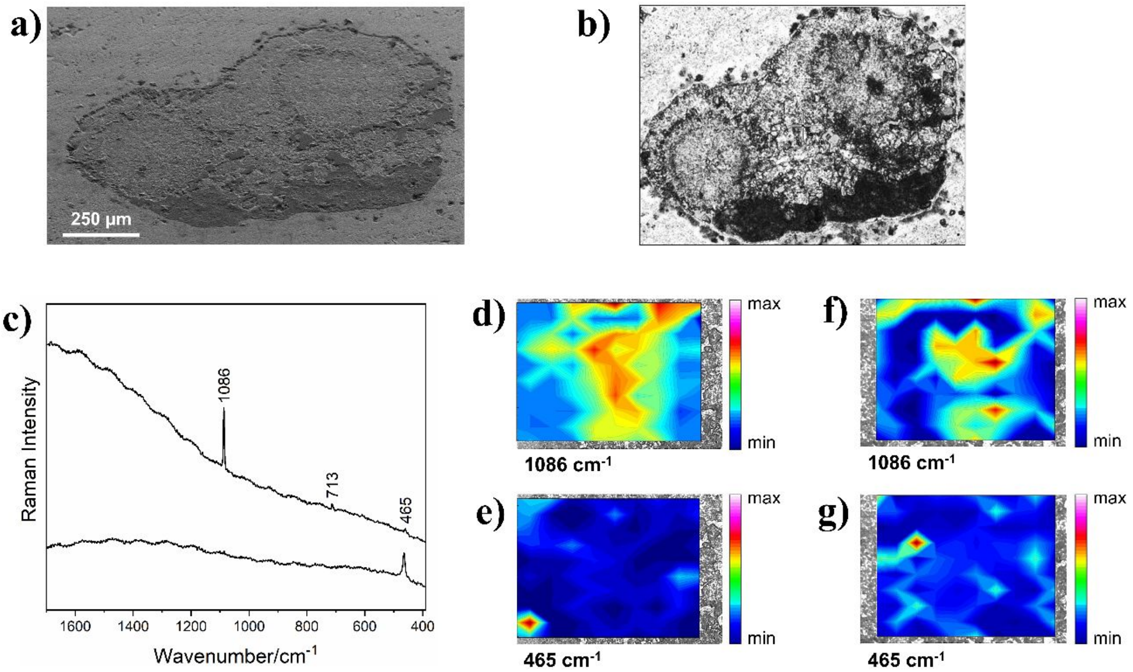

Figure S2. Raman analyses of the petrograph thin section from 5163 m depth. a) SEM at 45º (180×) of carbonate spherulite, b) Reflected light imaging of the carbonate spherulite (1180 x 1500 μm), 50×, and c) Selected Raman spectra obtained from mapping performed in the two spheres, with excitation laser line in 632.8 nm, 9 mW, 3 co-additions, 15s. Chemical maps obtained from 100 points in each of the two spheres: on the left side (430 × 600μm) and the right side (445 × 555μm). Respective integration of Raman bands d), f) 1086 cm^{-1} (CO_3^{-2}) and e), g) 464 cm^{-1} (SiO_2). The color scale indicates the magnitude of the integrated band area.

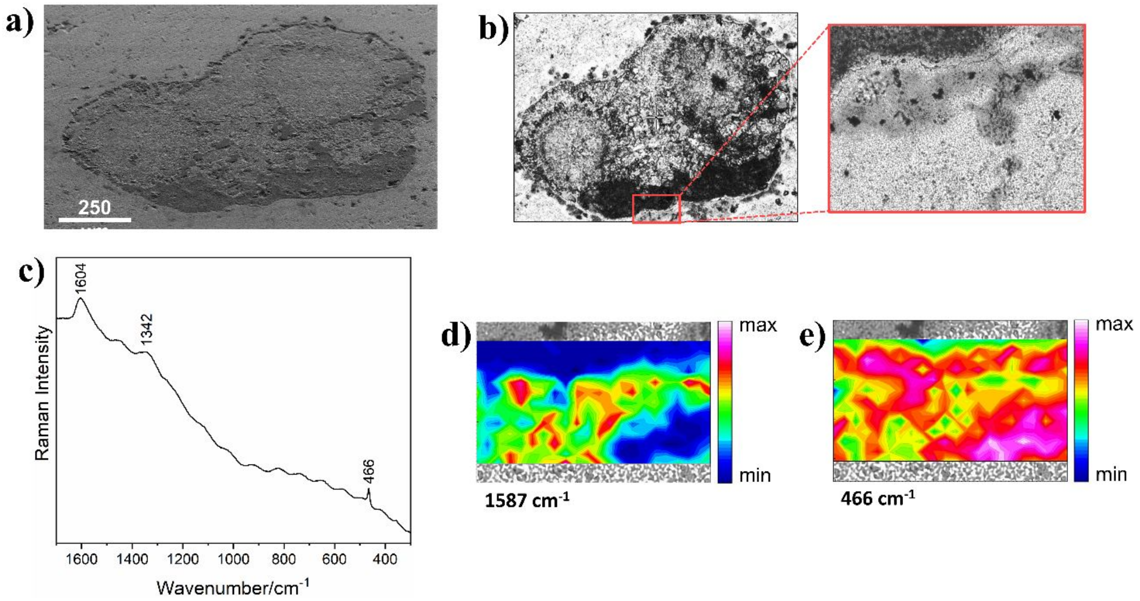

Figure S3. Raman analyses of the petrograph thin section from 5163 m depth. a) SEM at 45º (180×), b) Reflected light imaging of the carbonate spherulite (1180 x 1500 µm) and silica sphere-like structures in the expanded image, 50×, c) Selected Raman spectrum from mapping in 40 points (100 × 100 µm) with excitation laser line in 532 nm, 1.3 mW, 2 co-additions, 10s, and d) and e) Chemical maps obtained by the integration of Raman band at 1587 cm^{-1} (G band), and 464 cm^{-1} (SiO_2), respectively. The color scale indicates the magnitude of the integrated band area.

a) b)

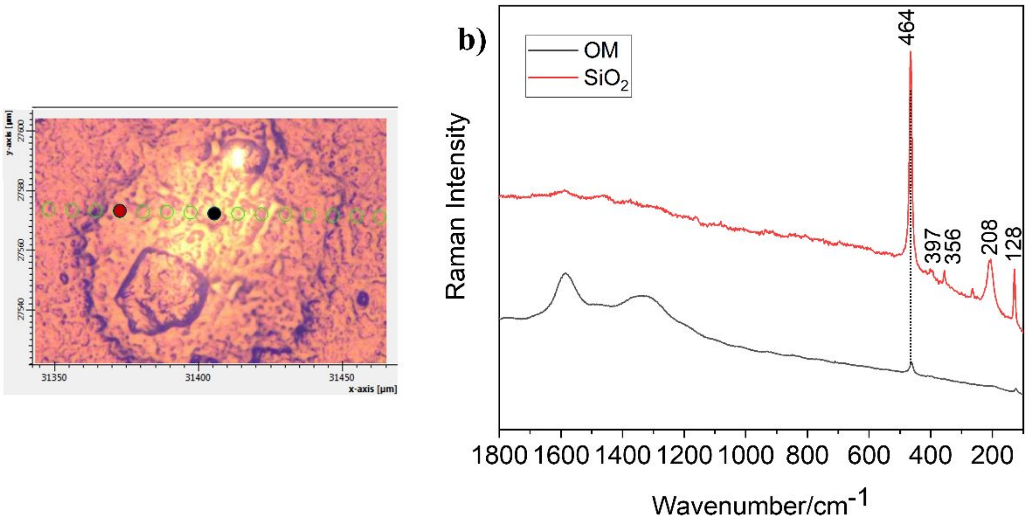

Figure S4. a) Reflected light imaging of the silica sphere-like structure (5188-C) showing in-line Raman mapping performed with 632.8 nm laser excitation; b) Selected Raman spectra from 15 points mapping (9 mW, 3 accumulations, 15 s, with spectral range of 1780-40 cm^{-1}, 3-5 cm^{-1} spectral resolution).

Table S1. Experimental parameters used for Raman spectral acquisition.

Depth (m)	Spectra code	Mapped area (μm²)/points[#]	Laser line (nm)	Laser power (mW)	Exposure time(s)	spectral range (cm^{-1})
5111	5111A	320×280 / 625	785	50	20	2630-60
	5111a1	6	532	1.3	20	1720-280
	5111a2	9	532	1.3	45	1720-280
	5111a3	4	532	1.3	45	1720-280
	5111a4	11	532	1.3	45	1720-280
	5111B	1	532	1.3	50	1720-280
5163	5163A	540×430 / 100	632.8	4.5	20	1780-390
	5163B	540×430 / 100	632.8	4.5	20	1780-390
	5163c	12×12 / 9	532	6.3	30	1720-280
	5163c	40×25 / 16	632.8	4.5	50	1780-390
	5163d	16×16 / 16	532	6.3	30	1720-280
	5163e	18×18 / 16	532	6.3	30	1720-280
	5163f	16×16 / 16	532	6.3	30	1720-280
	5163f	14×14 / 16	632.8	4.5	30	1780-390
	5163h	100×100 / 40	532	3.2	20	2700-50
5188	5188a	25×25 / 16	532	12.2	30	1720-280
	5188a	67×67 / 144	632.8	9	30	3500-40
	5188b	33×33 / 16	532	6.3	20	1720-280
	5188b	30×35 / 36	632.8	9	30	3500-40
	5188b	265×70 / 1600	632.8	9	56	1780-390
	5188c	163×200 / 1600	632.8	9	40	1780-390
	5188c	28×28 / 25	632.8	9	60	3500-40
	5188c	15	632.8	9	45	3500-40
	5188c	30×45 / 25	532	6.3	30	1720-280

#Number of random and mapped points.

Table S2. Deconvoluted Raman band assignments and spectral parameters (532 and 632.8 nm) calculated from organic matter identified in the silica sphere-like structures.

Depth/m	Spectral code	D1-wavenumber/cm^{-1}	G-wavenumber/cm^{-1}	G-FWHM/cm^{-1}	RBS/cm^{-1}	I_{D1}/I_G	A_{D1}/A_G	Estimated %Ro	R^2
5111	5111a2	1340.39	1605.53	71.94	265.13	2.07	3.00	0.62	0.992664
	5111a2	1336.64	1607.60	67.42	270.96	1.23	1.63	0.66	0.993496
	5111a4	1344.70	1603.74	81.70	259.05	1.04	1.49	0.57	0.999213
	5111a2	1342.31	1607.66	66.45	265.35	1.62	2.83	0.67	0.998138
	5111b	1343.06	1604.62	59.38	261.56	1.34	1.73	0.81	0.995868
	5111a1	1352.17	1603.91	78.44	251.73	0.86	1.23	0.58	0.999114
	5111b	1343.29	1604.41	65.28	261.13	1.20	1.57	0.69	0.998522
5163	5163e	1342.31	1604.40	58.32	262.09	1.18	1.95	0.84	0.997208
	**5163f**	**1372.85**	**1594.55**	**72.63**	**221.70**	**0.95**	**1.05**	**0.59**	0.998274
	5163f	1338.49	1588.92	72.90	250.43	0.89	1.25	0.61	0.999648
	5163f1	1341.43	1602.56	61.78	261.13	1.00	1.62	0.75	0.998419
	5163c	1343.49	1594.18	79.19	250.69	1.11	1.72	0.58	0.999521
	5163d	1342.99	1590.79	80.56	247.80	1.03	1.31	0.57	0.996859
5188	5188a	1333.02	1579.48	112.67	246.46	1.33	1.36	0.54	0.997465
	5188a	1327.77	1578.17	72.54	250.40	1.16	1.19	0.61	0.999242
	5188a	1329.71	1577.10	80.72	247.38	1.23	1.29	0.57	0.995436

	5188b	1330.36	1572.40	75.64	242.05	1.36	1.75	0.59	0.997349
	**5188b**	**1366.94**	**1581.03**	**72.54**	**214.09**	**0.79**	**1.77**	**0.94**	0.997008
	**5188b**	**1377.65**	**1587.40**	**79.67**	**209.75**	**0.78**	**0.91**	**0.69**	0.998555
	**5188b**	**1379.82**	**1589.31**	**84.83**	**209.49**	**0.79**	**1.00**	**0.56**	0.998047
	**5188c**	**1309.39**	**1583.87**	**63.41**	**274.48**	**0.99**	**2.07**	**1.39**	0.999250
	**5188a**	**1328.14**	**1603.16**	**63.46**	**275.02**	**1.42**	**2.36**	**1.39**	0.999310
	**5188a**	**1336.46**	**1590.99**	**78.50**	**254.53**	**1.27**	**2.59**	**0.73**	0.999075
	**5188a**	**1322.00**	**1597.66**	**61.50**	**275.66**	**1.27**	**1.89**	**1.51**	0.993218
	5188a	1328.22	1579.56	68.64	251.34	1.43	1.62	0.65	0.999159
	5188a	1332.62	1580.23	77.53	247.60	1.40	1.71	0.58	0.998809
	5188a	1336.15	1586.69	73.49	250.54	1.09	1.24	0.60	0.998599
	5188b	1331.01	1577.64	97.79	246.63	1.39	1.40	0.54	0.998334
	5188c	1339.13	1607.07	71.33	267.94	1.47	2.09	0.62	0.996757
MAX		1379.82	1607.66	112.67	275.66	2.07	3.00	1.13	0.999648
MIN		1309.39	1572.40	58.32	209.49	0.78	0.91	0.44	0.992664
AVERAGE		1341.12	1592.57	74.15	251.45	1.20	1.68	0.71	0.997674
STANDARD DEVIATION		15.94	11.38	11.42	18.07	0.28	0.52	0.27	0.001907

Data in bold were obtained with 632.8 nm laser excitation.

The correlation between %Ro and G-FWHM is represented by an exponential function (Equations 1 and 2) previously described in Barberes, G. A., et al. *J. Raman Spectr*. **2025**; 0:1-12. https://doi.org/10.1002/jrs.6836. The exponential relationships for 532 nm and 632.8 nm are expressed in Equations 1 and 2, respectively.

Eq. 1

$$\%Ro = \ 0.53901 + 98.46014\ e^{(-G\ FWHM/10.04782)}$$

Eq. 2

$$\%Ro = \ 0.40016 + 1360.60617\ e^{(-G\ FWHM-G/8.17184)}$$
